# Ancient drainage networks mediated a large‐scale genetic introgression in the East Asian freshwater snails

**DOI:** 10.1002/ece3.6523

**Published:** 2020-07-14

**Authors:** Osamu Miura, Misako Urabe, Hideaki Mori, Satoshi Chiba

**Affiliations:** ^1^ Faculty of Agriculture and Marine Science Kochi University Nankoku Japan; ^2^ Department of Ecosystem Studies School of Environmental Science The University of Shiga Prefecture Hikone Japan; ^3^ Japan Wildlife Research Center Tokyo Japan; ^4^ Division of Ecology and Evolutionary Biology Graduate School of Life Sciences Tohoku University Sendai Japan

**Keywords:** ancient lake, ddRAD, mitochondrial introgression, paleodrainage, Semisulcospira

## Abstract

Biogeography and genetic variation of freshwater organisms are influenced not only by current freshwater connections but also by past drainage networks. The Seto Inland Sea is a shallow enclosed sea in Japan, but geological evidence showed that a large freshwater drainage had intermittently appeared in this area between the late Pliocene and Pleistocene. Here, we demonstrated that this paleodrainage greatly affected the genetic variation of the East Asian freshwater snails, *Semisulcospira* spp. We found that the mtDNA haplotypes originated in the Lake Biwa endemic *Semisulcospira* species at the upstream side of the paleodrainage were frequently observed in the riverine *Semisulcospira* species at its downstream side. The genome‐wide DNA and morphological analyses consistently showed that there was no clear evidence of nuclear introgression between the Lake Biwa endemics and riverine species. These results suggest that the large paleodrainage had facilitated mitochondrial introgression and had broadly spread the introgressed mtDNA haplotypes to its downstream region around the Seto Inland Sea. Our study highlights the role of paleodrainages in shaping the genetic variation of freshwater organisms.

## Graphical abstract

The pattern of biogeography and genetic variation in modern freshwater species should reflect not only current but also ancient landscapes. We found that the paleo‐Setouchi drainage in Japan facilitated mitochondrial introgression between lake and riverine species and had broadly spread the introgressed mtDNA haplotypes to its downstream region. Our study highlights the role of paleodrainages in shaping the genetic variation of freshwater species.

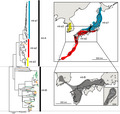

## 1 INTRODUCTION

The pattern of biogeography and genetic variation in modern freshwater species should reflect not only the current landscapes but also the ancient geography (Hewitt, 2000; Petit et al., 2003). The historical change in drainage networks is a fundamental factor in shaping the distribution and genetic structure in obligate freshwater organisms, due to their limited dispersal opportunities among drainage systems. The drainages could temporally be extended by seawater regression and be contracted by transgression (de Bruyn et al., 2013; Thomaz, Malabarba, Bonatto, & Knowles, 2015; Thomaz, Malabarba, & Knowles, 2017; Unmack, Hammer, Adams, Johnson, & Dowling, 2013; Wu, Tsang, Chen, & Chu, 2016; Yan et al., 2013). In addition, orogenesis can rearrange the drainage networks (Yan et al., 2013). These historical connections and dissociations of freshwater drainages should have changed the migration pattern of freshwater organisms and, therefore, intimately determine the distribution and genetic diversity of freshwater organisms.

**FIGURE 1:**
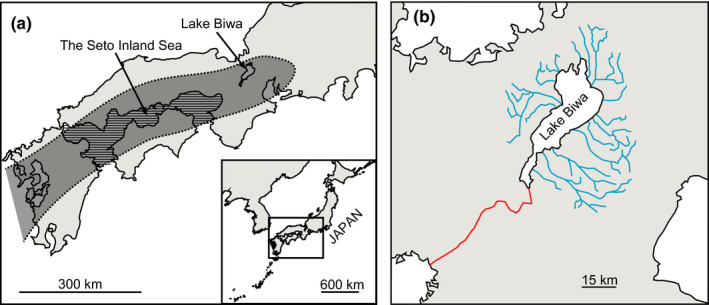
*The map of the Seto Inland Sea and Lake Biwa (a). The shaded area indicates the hypothetical location of the paleo‐Setouchi drainage intermittently appeared between late Pliocene and Pleistocene. The area with horizontal lines indicates the Seto Inland Sea. The current drainage of Lake Biwa (b). The blue lines indicate the rivers flowing into Lake Biwa, and the red line indicates the river flowing from Lake Biwa*. [Color figure can be viewed at wileyonlinelibrary.com]

The Seto Inland Sea is the largest enclosed sea in Japan and has historically experienced substantial changes in its topography and environment (Itihara, 1996; Kuwashiro, 1959). The region around the Seto Inland Sea is currently filled with seawater, but it had been a plain in the late Miocene (Itihara, 2001). Gentle block tilting during the late Pliocene and early Pleistocene produced a basin around the Seto Inland Sea area (the Setouchi Basin), which facilitated the formation of a large paleodrainage that is called the paleo‐Setouchi drainage (Figure 1a). The geological evidence exhibited that the paleodrainage included the ancient Lake Biwa on the upstream side and flowed west through the Setouchi Basin and Kyushu Island to the East China Sea (Itihara, 1996, 2001). The block tilting became active, and seawater had entered in this basin after the middle Pleistocene. However, the successive regression and transgression by cyclical climatic changes during the Quaternary period produced temporal freshwater drainage in this basin several times because the Seto Inland Sea is a shallow sea with the current average depth of 31 m. For example, a large part of the Setouchi Basin should be exposed and had converted into terrestrial and freshwater environments due to 140 m lowering of sea level during the Last Glacial Maximum about 20,000 years ago (Kuwashiro, 1959; Yashima, 1994). These geological events should rearrange the populations of freshwater organisms around the Setouchi Basin and affect their genetic diversity. Specifically, since the river flowing from Lake Biwa currently end up at the Seto Inland Sea (Figure 1b), freshwater organisms in Lake Biwa hardly achieve gene flow at a broad spatial scale. However, when the large paleodrainage had formed around the Setouchi Basin, the river flowing from Lake Biwa was likely to be connected with other drainages around the basin, which may facilitate the connection of freshwater populations among the lake and rivers around the basin.

*Semisulcospira* is the freshwater snail genus broadly distributed in East Asia (Davis, 1969). *Semisulcospira* is viviparous with no planktonic larval stage and thus has limited dispersal ability. Further, *Semisulcospira* is an obligate freshwater genus, which cannot traverse terrestrial or marine environments. Therefore, the *Semisulcospira* snails are ideal organisms to investigate the role of paleodrainages on the gene flow among the local populations. Eighteen species of *Semisulcospira* are reported in Japan, while one species, *Semisulcospira libertina,* is the most common, being distributed in all regions in Japan (Kihira, Matsuda, & Uchiyama, 2003). In this study, we investigate mtDNA and genome DNA variations of *Semisulcospira* spp. to evaluate how and what extent the paleodrainage had affected the genetic variation of *Semisulcospira* spp. The entire configuration of the paleo‐Setouchi drainage is still unclear, and the drainage might have connected to the river systems in the continental region (Itihara, 2001). We, therefore, collected the snails from broad geographical areas in Japan and Korea to evaluate the significance of the paleodrainage in shaping the genetic variation of the *Semisulcospira* snails.

## 2 MATERIALS AND METHODS

### 2.1 Sample collections

**FIGURE 2:**
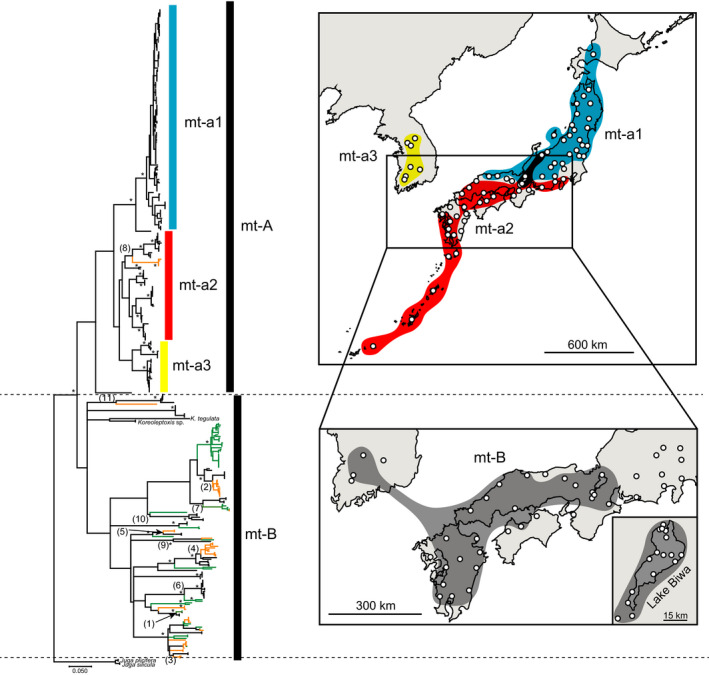
The mitochondrial phylogeny and geographical distribution of the mitochondrial clades of *Semisulcospira* spp. The asterisks near the major nodes of the phylogenetic tree indicate high bootstrap supports (>95). The black lines are individuals of the* S. libertina* group (*n* = 299), orange lines are individuals in the* S*. (*B*.) *decipiens* group (*n* = 47), and green lines are individuals of the* S*. (*B*.) *habei* group (*n* = 53). The numbers in parentheses indicate the nodes used for the divergence time estimation (see Table 1). The upper map shows the distribution of the three subclades in mt‐A clade (blue for mt‐a1, red for mt‐a2, and yellow for mt‐a3), while the lower map shows the distribution of mt‐B clade (gray). The detailed sampling locations for the Lake Biwa endemics are shown at the lower right. [Color figure can be viewed at wileyonlinelibrary.com]

Approximately four hundred individuals of *Semisulcospira* spp. were collected at 82 sites in Japan and Korea (Figure 2 and Appendix S1). The most common species, *S. libertina*, was collected from 67 sites. *Semisulcospira reiniana* and *S. kurodai* were collected from four and one sites, respectively. The endemic species group in Lake Biwa (the *Semisulcospira* (*Biwamelania*) *habei* group and *Semisulcospira* (*Biwamelania*) *decipiens* group, see Miura, Urabe, Nishimura, Nakai, & Chiba, 2019) were obtained from 16 sites in Lake Biwa and its drainage. We identified these species based on adult shell features and the number and shape of embryos following the procedures of Davis (1969) and Watanabe and Nishino (1995). However, the taxonomy of *Semisulcospira* is currently confused. For example, although *S. reiniana* has a distinct shell morphology, it genetically belongs to a clade of *S. libertina* (Miura et al., 2019). Further, the species in the Lake Biwa endemic groups have very similar genome DNA sequences (Miura et al., 2019). We, therefore, use the species groups (the *S. libertina* group, *S*. (*B*.) *habei* group, and *S*. (*B*.) *decipiens* group), rather than morphospecies, as operational units in this study. Snails were either fixed in 95% ethanol, stored at −30°C or both for molecular analyses. We isolated genome DNA using a modified CTAB procedure described by Miura, Torchin, Bermingham, Jacobs, and Hechinger (2012). Some of those samples were also used in the prior studies (Miura, Köhler, Lee, Li, & Foighil, 2013; Miura et al., 2019).

### 2.2 Mitochondrial DNA analyses

A total of 406 *Semisulcospira* spp. and related species were used for sequence analysis (Appendix S1). We analyzed mtDNA encoding the cytochrome oxidase *c* subunit I (COI) gene to identify major lineages and their phylogeographic patterns. The primer pair used in this study was COI‐bf (5′‐GGGGCTCCTGATATAGCTTTTCC‐3′) (Miura, Torchin, Kuris, Hechinger, & Chiba, 2006) and COI‐6 (5′‐GGRTARTCNSWRTANCGNCGNGGYAT‐3′) (Shimayama et al., 1990). Further, we developed an inner primer pair, semlong (5′‐GGAGCACCAGATATAGCTTTCCC‐3′) and semR (5′‐AAGTGYGCTTTTGTTCATCGRG‐3′). The second primer pair was used when the target DNA was hardly amplified by the first primer pair. The DNA was amplified in 35 PCR cycles under the following conditions: denaturing at 94°C for 60 s, annealing at 45°C (50°C for the inner primer pairs) for 60 s, and extension at 72°C for 90 s. The 35 cycles were preceded by an initial denaturing step at 94°C for 1 min and followed by a final extension of 72°C for 7 min. The PCR products were purified and sequenced from both directions using automated sequencers. Sequences analyzed in this study were deposited in GenBank/DDBJ (accession nos. KX080149–KX080549 and LC500664–LC500669). We ensured the validity of sequences by inspecting the sequence chromatograms and by checking the quality scores of each base using Geneious 6.1.6 (Kearse et al., 2012). Sequences were aligned by ClustalW, implemented in Geneious for further phylogenetic analyses. Phylogenetic trees were constructed using the maximum likelihood (ML) algorithms. We searched for the best evolutionary models using MEGA 7.0.18 (Kumar, Stecher, & Tamura, 2016); the HKY + G + I model was selected. The ML analysis was also conducted using MEGA 7.0.18. Node robustness was assessed using bootstrapping, and 1,000 replicates. We selected *Juga plicifera*, *Juga silicula*, *Koreoleptoxis* sp., and *Koreoleptoxis tegulata* as outgroups for the COI phylogeny, based on the earlier studies (Köhler, 2016; Lee, Hong, Kim, & Foighil, 2007; Miura et al., 2019; Strong & Köhler, 2009).

### 2.3 Genome DNA analyses

Because of the unusual mitochondrial evolution in *Semisulcospira* (Köhler, 2017) and related snails (Whelan & Strong, 2016), other independent measurements are necessary to evaluate the phylogenetic relationship of *Semisulcospira* spp. We, therefore, analyzed genome‐wide DNA variations in *Semisulcospira* spp. We used 39 *S*. *libertina* genome DNA samples; these samples were the subset of the samples used in the mtDNA analysis (Appendix S2). We analyzed the genome‐wide DNA variations using a double digest restriction site‐associated DNA library (ddRAD) sequencing technique, as described by Peterson, Weber, Kay, Fisher, and Hoekstra (2012) with a slight modification (Miura et al., 2019). Briefly, the extracted DNA from each individual was further purified using a NucleoSpin gDNA Clean‐up Kit (Macherey‐Nagel) with the addition of RNase A. Approximately 30 ng of DNA was digested using two restriction enzymes (*Eco*RI and *Msp*I). P1 and P2 adapters from Peterson et al. (2012) were ligated to the DNA fragments of each individual. The ligated samples were multiplexed and purified using a NucleoSpin gDNA Clean‐up Kit. An E‐gel size select agarose gel (Invitrogen, CA) was used to collect 300‐ to 350‐bp DNA fragments. We amplified the DNA fragments in 8 single PCR reactions. The PCR products were combined and cleaned using E‐gel size select agarose gel and the NucleoSpin gDNA Clean‐up Kit. The constructed DNA library was sent to Genome Quebec Innovation Center and sequenced using Illumina HiSeq 2000 single‐end sequencing, yielding maximum read lengths of 100 bp.

Raw sequence reads were processed using pyRAD 3.0.66 (Eaton, 2014). We integrated these sequence reads with the genome DNA sequences published in Miura et al. (2019) (DDBJ accession nos. DRA004774, DRA005712, and DRA007043). In total, the sequence reads from 143 individuals, including 28 from the* S*. (*B*.) *habei* group, 29 from the *S*. (*B*.) *decipiens* group, 78 from the* S. libertina* group, and 8 outgroups, were used for the subsequent analyses (Appendix S2). Sequences were demultiplexed using their sample‐specific barcode without allowing any mismatches. The restriction site and barcode were removed from each sequence. A nucleotide base with a FASTQ quality score less than 20 was replaced with N. Sequences having more than 5% Ns were discarded. Sequences within each sample were clustered using VSEARCH (Rognes, Flouri, Nichols, Quince, & Mahé, 2016) with an 85% similarity threshold, following the pyRAD SE ddRAD tutorial. Within‐sample clusters with fewer than 10 sequences were excluded to ensure accurate base calls. Consensus sequences were created based on the clusters with consideration of the error rate and heterozygosity. Consensus sequences from all samples were clustered using the same similarity threshold that was applied in the within‐sample clustering. The resulting across‐sample clusters were aligned with MUSCLE (Edgar, 2004). Any clusters having more than 5% shared polymorphic sites were discarded because a shared heterozygous site across many samples likely represents a clustering of paralogs (Hohenlohe, Amish, Catchen, Allendorf, & Luikart, 2011). Clusters shared among fewer than 50 individuals were excluded (the minimum taxon coverage = 50), and the remaining clusters were treated as ddRAD loci.

The ddRAD sequences were concatenated into a single sequence alignment by an output function in pyRAD. Phylogenetic analysis was conducted by maximum likelihood (ML) algorism, using RAxML 8.0.20 (Stamatakis, 2014) with general time‐reversible and gamma model (GTR + G). Node robustness was assessed using bootstrapping and 100 replicates. *J. plicifera*, *J. silicula*, *Koreoleptoxis* sp., and *K. tegulata* were selected as an outgroup. We further estimated coancestry matrix to characterize genetic differentiation and admixture pattern, using the SNPs extracted from ddRAD loci. The coancestry values were calculated in fineRADstructure (Malinsky, Trucchi, Lawson, & Falush, 2018).

### 2.4 Estimation of divergence time

To determine when the gene flow (mitochondrial introgression) occurred among the populations in the currently separated drainage systems, we estimated divergence time using mitochondrial DNA variations and a molecular clock. The oldest fossil species that is endemic to Lake Biwa is recorded from the Ueno and Iga Formation in the Kobiwako Group and Kameyama Formation in the Tokai Group (3.9–3.2 million years ago [MYA] [Matsuoka, 1985, 2001]). This fossil species, *Semisulcospira* (*Biwamelania*) *praemultigranosa*, is the prospective earliest stem lineage of the Lake Biwa endemic snails (Matsuoka, 1985; Miura et al., 2019). Therefore, we set the split age of mt‐B at 3.9 MYA, with the assumption of the Lake Biwa origin of mt‐B. We selected eleven pairs of *S. libertina* and the Lake Biwa endemics, which are suspicious for mitochondrial introgression (see the numbered nodes in Figure 2) and estimated the age of their divergence. Because the relative rate test rejected equal evolutionary rate throughout the tree (*p* < .01), the divergence time was estimated based on uncorrelated relaxed lognormal clock implemented in BEAST v. 2.5.1 (Bouckaert et al., 2014). An exponential prior distribution was used for the split of mt‐B. The Yule speciation model was used as a tree prior. The same evolutionary model, as the prior phylogenetic analysis, was used in the analysis. The analysis was run for 100 million generations, sampled every thousand steps, and the first thousand samples were discarded as burn‐in. To check for convergence and to visualize the results, we used TRACER v. 1.6 and FIGTREE v. 1.4.2 (Drummond & Rambaut, 2007).

### 2.5 Morphometrics

To evaluate whether introgressive hybridization affected the morphology of the *Semisulcospira* snails, we photographed 188 shells of the* S. libertina* group and 28 shells of the *S*. (*B*.) *habei* group and 26 shells of the *S*. (*B*.) *decipiens* group using a stereomicroscope with an Olympus DP 26 camera system at 0.21× magnification (see Appendix S1). Note that *S*. (*B*.) *nakasekoae* was excluded from the analysis because it did not have a sufficient number of whorls for the morphological comparison (>3 wholes). Twelve landmarks describing the shell shape of each snail were digitized from images using TpsDig2 (available at http://life.bio.sunysb.edu/morph/). We set the landmarks on the edge of sutures and aperture (Figure 5). The criteria for choosing the location of the landmarks were their general ease in identification (Madeira, Alves, Mesquita, Silva, & Paula, 2012). Procrustes transformation of the coordinate was done with PAST 3 (Hammer, Harper, & Ryan, 2001), followed by relative warp analysis to summarize shape variation in two‐dimension graphics. We statistically evaluated the morphological differences among *S. libertina* with mt‐A haplotypes, *S. libertina* with mt‐B haplotypes, the *S* (*B*.) *habei* group, and the *S*. (*B*.) *decipiens* group using a pairwise MANOVA with Bonferroni corrections for multiple comparisons.

## 3 RESULTS

Based on 812 bp of the COI gene, 191 haplotypes were identified. There were two largely distinct clades in *Semisulcospira* in Japan and Korea (Figure 2). Mt‐A was numerically predominant and was broadly distributed in Japan and Korea. Our dataset showed that this clade involved three subclades (mt‐a1, mt‐a2, and mt‐a3). Mt‐a1 mainly occurred in northeastern Japan, while mt‐a2 occurred in southwestern Japan. In addition, mt‐a3 was exclusively distributed in Korea (Figure 2). The geographical distributions of these subclades were mostly separated, though mt‐a1 and mt‐a2 co‐occurred in a few locations near the boundary of their distribution ranges (Figure 2). On the other hand, mt‐B involved highly diverged lineages and was distributed only around the Seto Inland Sea area and a few locations in Korea (Figure 2). Importantly, most of the endemic snails in Lake Biwa have mitochondrial DNA haplotypes that belong to mt‐B, except for a few haplotypes that belong to mt‐a2.

**FIGURE 3:**
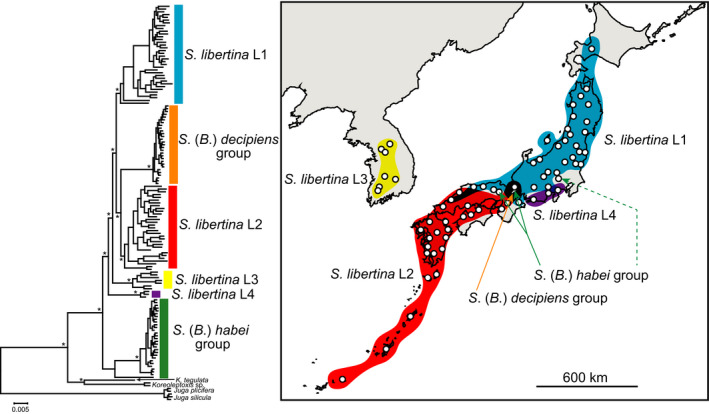
The genome DNA phylogeny and geographical distribution of the genome DNA clades. The asterisks near the major nodes of the phylogenetic tree indicate high bootstrap supports (>95). The colors indicate the distribution area of each *Semisulcospira* clade (blue for *S. libertina* L1, red for *S. libertina* L2, yellow for *S. libertina* L3, purple for *S. libertina* L4, orange for the *S*. (*B*.) *decipiens* group, and green for the *S*. (*B*.) *habei* group). The distribution area of the *S*. (*B*.) *decipiens* group and *S*. (*B*.) *habei* group was manly in the Lake Biwa drainage. The left side of the green bifurcated arrow pointed out the sampling location of *S. kurodai*. The green dotted arrow indicates the sampling location of the *S. libertina* individual that genetically belongs to the *S*. (*B*.) *habei* group. [Color figure can be viewed at wileyonlinelibrary.com]

**FIGURE 4:**
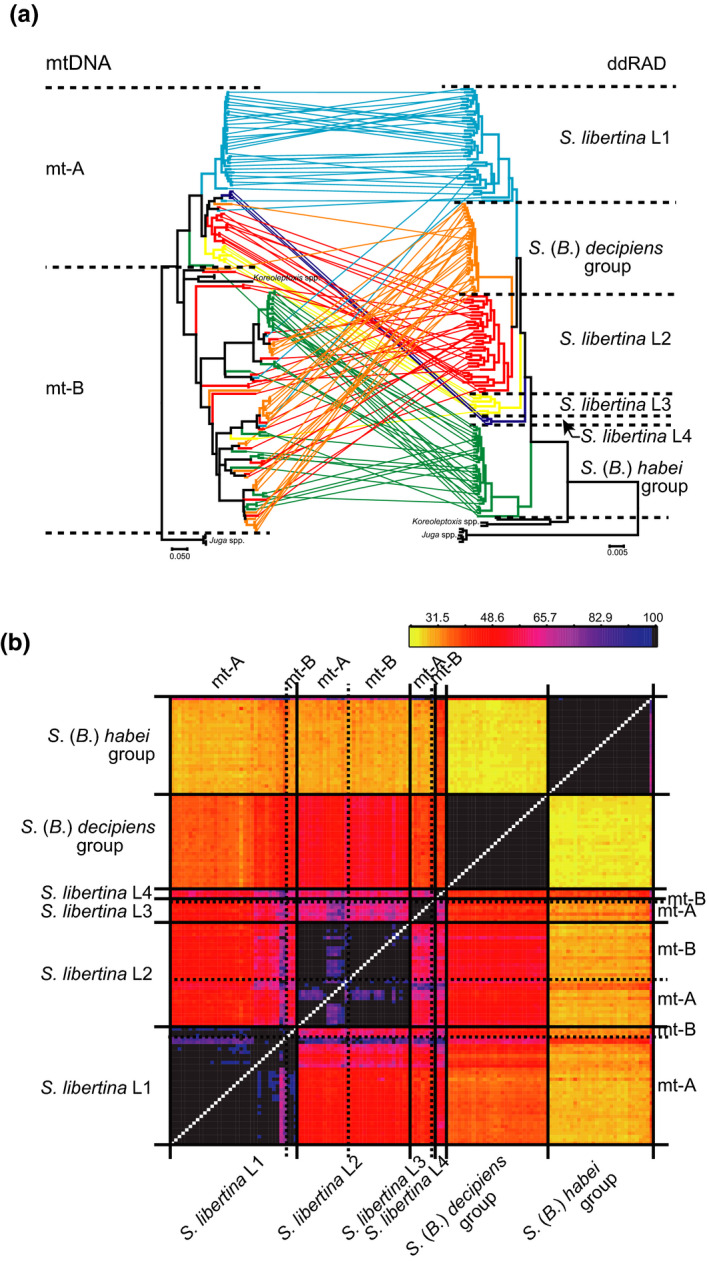
The evidence of mitochondrial introgression between riverine and Lake Biwa endemic species. Comparison between the mitochondrial and genome DNA phylogenies (a). The same individuals in both trees are joined by lines. The color indicates the genome DNA clades (blue for *S. libertina* L1, red for *S. libertina* L2, yellow for *S. libertina* L3, purple for *S. libertina* L4, orange for the *S*. (*B*.) *decipiens* group, and green for the *S*. (*B*.) *habei* group). The coancestry matrix among the individuals from each clade (b). The genome DNA clades were shown at the left and bottom, and the mitochondrial clades for *S. libertina* (mt‐A or mt‐B) were shown at the right and top. [Color figure can be viewed at wileyonlinelibrary.com]

We obtained 0.4 to 15.3 million reads for each individual after demultiplexing the Illumina HiSeq raw dataset. The average number of clusters greater than nine sequences was 18,201; the average coverage achieved per individual per loci was 50.6 (Appendix S2). There were 10,008 loci with a total alignment length of 960,071 bp, and the dataset had 41% of missing data. The ML tree based on the concatenated ddRAD sequences is shown in Figure 3. There were six clades in the ML tree (*S. libertina* L1–L4, *S*. (*B*.) *decipiens* group, and *S*. (*B*.) *habei* group). Of the six clades, the geographical distributions of three clades (*S. libertina* L1–L3) corresponded with those of the mitochondrial clades, mt‐a1–mt‐a3. The clade *S. libertina* L1 was distributed in northeastern Japan, and most individuals that have mtDNA haplotype mt‐a1 belonged to this clade. The clade *S. libertina* L2 was distributed in southwestern Japan, and the majority of individuals that have mtDNA haplotype mt‐a2 belonged to this clade. In addition, the clade *S. libertina* L3 was distributed in Korea, where almost all individuals have mtDNA haplotype mt‐a3. Although most individuals with mt‐a2 mtDNA haplotypes belonged to the genome clade *S. libertina* L2, the individuals that were distributed around Tokai districts formed a distinct genome clade *S. libertina* L4. The *S*. (*B*.) *habei* group and *S*. (*B*.) *decipiens* group were mainly observed in Lake Biwa and its drainage. However, we found that one individual of *S. libertina* at a distant location from Lake Biwa belonged to the *S*. (*B*.) *habei* group in the genome DNA phylogeny (see the dotted arrow in Figure 3). Further, this individual had a unique mtDNA haplotype that was located at the base of mt‐A (Figures 2 and 4). Since we lack an adequate understanding of the evolutionary origin of this individual, we do not explore it in detail in this study.

The discordances between the mitochondrial and genome DNA phylogenies were observed (Figure 4a). The mt‐B haplotypes were dominant in Lake Biwa, but these haplotypes also appeared in the regions around the Seto Inland Sea and southern Korea (Figure 2). In these regions, the *S. libertina* individuals with mt‐A haplotypes and those with mt‐B haplotypes often co‐occurred at the same or neighboring locations. Despite the considerable divergence of their mtDNA haplotypes, we found that these individuals in geographically close locations were closely related in the genome DNA phylogeny (Figure 4a). Two *Koreoleptoxis* species included in mt‐B were located outside of the *Semisulcospira* clade in the genome DNA phylogeny (Figure 3). The basal placement of *Koreoleptoxis* at mt‐B clade in the mitochondrial phylogeny (Figure 2) is likely to be artificial and flawed by long‐branch attraction effect (Bergsten, 2005).

The coancestry matrix also exhibited six genetically distinct groups, supporting the clustering pattern in the genome DNA phylogeny (Figure 4b). Our results showed that *S. libertina* individuals with mt‐A and those with mt‐B haplotypes exhibited very similar coancestry patterns. The results further showed that there was no clear genomic contribution from the Lake Biwa endemics to *S. libertina* (with mt‐B haplotypes) (Figure 4b), despite their mitochondrial similarity. The divergence time estimates demonstrated that the mitochondrial introgression between *S. libertina* and the Lake Biwa endemics occurred between 1.93 and 0.19 MYA (Table 1).

**TABLE 1:** The estimated ages of the mitochondrial introgression events between *S. libertina* and the Lake Biwa endemics. The node numbers correspond to the nodes of the mtDNA tree displayed in Figure 2

Node number	Estimated age (MYA)	95% HPD (MYA)
(1)	0.19	0.09–0.30
(2)	0.37	0.20–0.54
(3)	0.45	0.26–0.65
(4)	0.47	0.28–0.67
(5)	0.6	0.37–0.88
(6)	0.69	0.43–0.97
(7)	0.7	0.45–0.99
(8)	1.05	0.62–1.35
(9)	1.41	0.93–1.92
(10)	1.78	1.15–2.24
(11)	1.93	1.31–2.63

**FIGURE 5:**
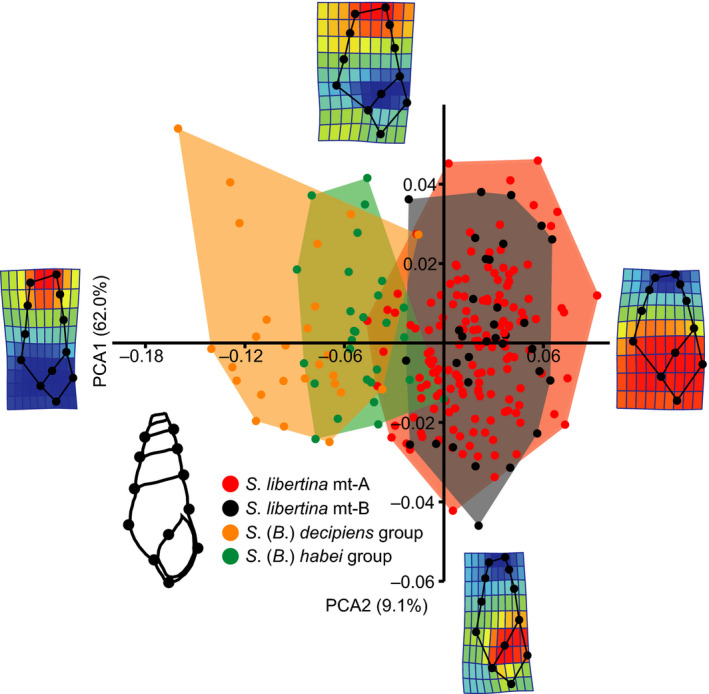
The relative warp analysis demonstrating the morphological difference among *S. libertina* and the Lake Biwa endemics. The eigenvalue for each axis was 0.0021 for PCA1 and 0.0003 for PCA2. The percentage variance explained by each axis is indicated in parentheses next to the axis label. The landmarks used in the morphological analysis are shown on the shell drawings at the lower left of the graph. The shape changes along the axes are shown with the wireframe and heatmap visualization: red for expansion and blue for contraction. [Color figure can be viewed at wileyonlinelibrary.com]

The morphometric analyses demonstrated that there were no significant morphological differences between *S. libertina* with mt‐A haplotypes and that with mt‐B haplotypes (MANOVA, *p* = .96, Table 2) (see also Figure 5). However, the morphologies of these *S. libertina* groups were significantly different from those of the *S*. (*B*.) *habei* group (MANOVA, *p* < .01, Table 2) and the *S*. (*B*.) *decipiens* group (MANOVA, *p* < .01, Table 2).

**TABLE 2:** The pairwise MANOVA test for shell shape comparisons among the *Semisulcospira* groups. The pairwise squared Mahalanobis distances are arrayed below the diagonal, whereas the Bonferroni‐corrected *p*‐values are arrayed above the diagonal

	*S. libertina* mt‐A	*S. libertina* mt‐B	*S*. (*B*.) *habei* group	*S*. (*B*.) *decipiens* group
*S. libertina* mt‐A		0.96	<0.01	<0.01
*S. libertina* mt‐B	1.33		<0.01	<0.01
*S*. (*B*.) *habei* group	15.24	19.18		<0.01
*S*. (*B*.) *decipiens* group	34.44	38.08	11.93	

## 4 DISCUSSION

We found that the clustering pattern based on mitochondrial DNA variations was largely concordant to that based on the genome DNA variations. However, there were apparent exceptions. The mitochondrial and genome DNA discordance was often observed in the populations around the Seto Inland Sea. The mtDNA haplotypes which were likely to be originated within the Lake Biwa endemics were broadly spread over the region around the Seto Inland Sea, while these snails had the genome DNA loci clustering with the local riverine species. These results suggest that the paleo‐Setouchi drainage mediated mitochondrial introgression between the Lake Biwa endemics and riverine species and facilitated the spread of the introgressed haplotypes to the downstream area of the paleodrainage. Our results demonstrated that ancient connections of populations through the paleodrainage strongly affected the geographical genetic variations of the freshwater snails.

Lee et al. (2007) first investigated the genetic variation of *Semisulcospira* spp. in Korea and found that there was a strange mitochondrial clade that contained phylogenetically divergent haplotypes. They suggested that this “enigmatic” divergent mitochondrial clade was originated due to ancestral polymorphisms or was captured through introgressive hybridization. Miura et al. (2013) demonstrated that the divergent mitochondrial clade in Korea historically migrated from Japan, potentially through hybridization between Korean and Japanese snails during Pleistocene Glacial Maxima when Korean Peninsula had been connected to the Japanese archipelago. Köhler (2016) showed that Lake Biwa is a potential reservoir for the divergent clade. We found that this divergent mitochondrial clade (= mt‐B) was distributed exclusively around Lake Biwa and the Seto Inland Sea area in Japan and at the southern part of the Korean Peninsula (Figure 2). Importantly, most of the divergent haplotypes observed around the Seto Inland Sea area and Korea exhibited a sister relationship with the haplotypes in the Lake Biwa endemics (Figure 2). This phylogeographic pattern suggests that the divergent mitochondrial haplotypes in mt‐B were originated in Lake Biwa, and these haplotypes had spread to the regions around the Seto Inland Sea and the south of Korean Peninsula.

The presence of the mt‐B haplotypes in *S. libertina* suggests the hybridization between *S. libertina* and the Lake Biwa endemics. We compared the mtDNA variation and genome DNA variation to understand whether the hybridization between *S. libertina* and the Lake Biwa endemics led to the formation of hybrid swamps, or it only resulted in mitochondrial introgression. We found that all individuals of *S. libertina* with mt‐B haplotypes belonged to either *S. libertina* L1–3 clades in the genome DNA phylogeny (Figure 4a). Further, there was no remarkable genome contribution from the Lake Biwa endemics to these *S. libertina* individuals based on the coancestry matrix (Figure 4b). These results suggest that the presence of mt‐B haplotypes in *S. libertina* is most likely explained by mitochondrial introgression.

The morphological analyses also support the mitochondrial introgression. Hybrid lineages are often morphologically intermediate between parental species (e.g., Wilson & Hebert, 1993). However, our morphological analyses demonstrated that there were no apparent differences in the shell morphology between *S. libertina* with mt‐A haplotypes and that with mt‐B haplotypes (Figure 5). In addition, the outline morphologies of *S. libertina* with mt‐B haplotypes were significantly different from either the *S*. (*B*.) *habei* or *S*. (*B*.) *decipiens* groups (Figure 5, see also Table 2). These results further provided supportive evidence of the mitochondrial introgression between *S. libertina* and the Lake Biwa endemics, without the introgression of nuclear genes that control morphological characters of *Semisulcospira* spp.

Geological studies indicate that the large paleodrainage, which presumably involved several lakes and rivers, had appeared around the Setouchi Basin in the past (Itihara, 1996, 2001) (see also Figure 1). This paleodrainage was first formed about 3 – 1 MYA. After this period, the Setouchi Basin had been covered by seawater (Itihara, 1996). However, successive regression and transgression due to glaciations between 1 MYA and 20,000 years ago (until the Last Glacial Maximum) intermittently restored a temporal paleodrainage in this area (Kuwashiro, 1959; Yashima, 1994). This paleodrainage had strongly affected the migration patterns and phylogeography of freshwater fishes in Japan (Takahashi et al., 2020; Takehana, Nagai, Matsuda, Tsuchiya, & Sakaizumi, 2003; Watanabe et al., 2010). We assumed that the Lake Biwa endemics extended their distribution range through the paleodrainage and transferred their mtDNA haplotypes to local *S. libertina* populations through mitochondrial introgression. Matsuoka and Kitabayashi (2001) found several Pliocene fossils in the subgenus *Biwamelania* (the endemic subgenus name for the Lake Biwa *Semisulcospira* snails) at Oita Prefecture in Kyushu, which is located at the western edge of the Setouchi Basin, supporting the temporal range expansion of the Lake Biwa endemics. We evaluated the time of the range expansion by estimating the time for the mitochondrial introgression between *S. libertina* and the Lake Biwa endemics. Our results exhibited that most of the estimated times ranged between 1.0 and 0.2 MYA (Table 1), suggesting the temporal paleodrainage formed during the glacial regression could facilitate mitochondrial introgression and spread mt‐B haplotypes around the Setouchi Basin. We also found that there was mitochondrial introgression in the reverse direction. The mt‐a2 haplotypes of *S. libertina* were introgressed to the Lake Biwa endemics about 1 MYA (the node [8] in Figure 2 and Table 1). Although some of the estimated ages were much older (1.9–1.4 MYA, Table 1), we consider that these time estimations can be biased to older times because of the insufficient sampling of closer sisters, or the paleodrainage formed before the glacial period might also have contributed to the introgressive hybridization between *S. libertina* and the Lake Biwa endemics.

The distribution of mt‐B haplotypes overlapped with that of mt‐a2 haplotypes (Figure 2). However, although mt‐a2 haplotypes were distributed in the Ryukyu islands, mt‐B haplotypes were absent from these islands. Geological studies indicate that Ryukyu and Kyushu islands had often connected in the past, but they have been completely separated after the formation of the deep channel about 1.7 MYA (Kitamura & Kimoto, 2004). The majority of the divergence time estimates indicated that mt‐B haplotypes moved outside Lake Biwa after the formation of the deep channel (Table 1), which could prevent the invasion of mt‐B into the Ryukyu islands. On the other hand, mt‐B haplotypes were often observed in the southern part of the Korean Peninsula (Figure 2). Since Tsushima Strait that separates Kyushu and the Korean Peninsula is shallow, the northern part of Kyushu and the southern part of the Korean Peninsula had been connected by glacial regressions at least three times (at 1.20, 0.63, and 0.43 MYA) (Kawamura, 1998; Taruno, 2010). The estimated times for mitochondrial introgression between *S. libertina* and the Lake Biwa endemics were roughly consistent with the ages of the land bridge formation (Table 1). Our results indicated that the paleo‐Setouchi drainage had connected to Korean drainage systems through the land bridge formed during the glacial regressions (see also Miura et al., 2013). The historical connection of Kyushu and the Korean Peninsula during the glacial period was also supported by phylogeographic studies of fishes (Tominaga, Nakajima, & Watanabe, 2016), insects (Park, Kitade, Schwarz, Kim, & Kim, 2006), and plants (Okaura, Quang, Ubukata, & Harada, 2007).

The high level of mtDNA variations observed in the *Semisulcospira* snails had been elusive, and several hypotheses were proposed to explain this enigmatic diversity (Köhler, 2016; Lee et al., 2007; Miura et al., 2013). Similar enigmatic mitochondrial variations were reported in the related family Pleuroceridae (Dillon & Frankis, 2004; Dillon & Robinson, 2009; Whelan & Strong, 2016). Our genome DNA analyses with the extensive geographical sampling of the *Semisulcospira* snails demonstrate that the divergent mtDNA haplotypes originated in Lake Biwa had spread to a broader region through the paleodrainage. Our results indicate that the high mitochondrial variations and the incongruence between mtDNA phylogeny and taxonomy in *Semisulcospira* are largely explained by ancient mitochondrial introgression between the Lake Biwa endemics and riverine species. Our study highlights that the current genetic structure in the *Semisulcospira* snails cannot be fully understood without considering the ancient geological events. However, there are still incomplete understandings of the mitochondrial evolution in *Semisulcospira*. Specifically, we lack the conceptual framework to explain (a) how the divergent mtDNA haplotypes have originated in Lake Biwa, (b) why only mtDNA haplotypes had been spread from Lake Biwa without the introgression of nuclear genome DNA, and (c) why only mt‐B haplotypes had migrated to the Korean Peninsula through the ancient land bridge without the migration of mt‐A haplotypes. Future studies with a more in‐depth investigation of mtDNA evolution in relation to the demographic history of *Semisulcospira* spp. may provide a clue to disentangle these complex evolutionary issues in the *Semisulcospira* snails.

The detailed hydrological network of the paleo‐Setouchi drainage is still ambiguous despite geological research efforts (Itihara, 1996, 2001; Kuwashiro, 1959; Yashima, 1994). Itihara (1996) suggested that the paleo‐Setouchi drainage had potentially connected to the huge drainages in China, such as the Yellow River and Yangtze River, at the continental shelf exposed in the past. Although it is not easy to evaluate this hypothesis since these paleodrainage connections are currently hidden under the sea, we consider that this hypothesis will still be testable using *Semisulcospira* as a biological marker. If we can find mt‐B haplotypes from the *Semisulcospira* individuals in the Chinese drainages, and they exhibit a sister relationship with the haplotypes of the Lake Biwa endemics, it will indicate the past connection between the paleo‐Setouchi drainage and the Chinese drainages. Therefore, future studies on genetic variations in the Chinese *Semisulcospira* spp. will be fruitful, which will reveal not only the evolutionary history of the *Semisulcospira* snails but also the geographical evolution of the East Asian region. Our study demonstrates that the immobile freshwater species such as *Semisulcospira* spp. preserve the trace of ancient drainage networks within their DNA and thus can provide valuable information to reconstruct the ancient geography of focal regions.

## CONFLICT OF INTEREST

The authors declare no conflict of interest associated with this manuscript.

## AUTHOR CONTRIBUTION

**Osamu Miura:** Conceptualization (lead); Data curation (lead); Formal analysis (lead); Funding acquisition (equal); Investigation (lead); Writing‐original draft (lead). **Misako Urabe:** Conceptualization (equal); Investigation (equal); Writing‐review & editing (equal). **Hideaki Mori:** Data curation (equal); Investigation (equal); Writing‐review & editing (equal). **Satoshi Chiba:** Conceptualization (equal); Funding acquisition (equal); Supervision (equal); Writing‐review & editing (equal).

## Supporting information

Appendix S1

Appendix S2

## Data Availability

Mitochondrial DNA dataset used in this study is available at GenBank/DDBJ database (accession nos: KX080149–KX080549 and LC500664–LC500669). Genome DNA dataset used in this study is available at DDBJ Sequence Read Archive (accession nos: DRA004774, DRA005712, DRA007043, and DRA008971). The alignment and the ML tree for the mtDNA data and genome‐wide DNA data are available from the Dryad depository (https://doi.org/10.5061/dryad.np5hqbzqh).
